# Metagenomics Shines Light on the Evolution of “Sunscreen” Pigment Metabolism in the *Teloschistales* (Lichen-Forming Ascomycota)

**DOI:** 10.1093/gbe/evad002

**Published:** 2023-01-12

**Authors:** Theo Llewellyn, Reuben W Nowell, Andre Aptroot, Marina Temina, Thomas A K Prescott, Timothy G Barraclough, Ester Gaya

**Affiliations:** Comparative Fungal Biology, Royal Botanic Gardens, Kew, Jodrell Laboratory, Richmond, TW9 3DS, UK; Department of Life Sciences, Imperial College London, Silwood Park Campus, Ascot, Berkshire, SL5 7PY, UK; Science and Solutions for a Changing Planet Doctoral Training Partnership, Grantham Institute, Imperial College London, South Kensington, London, SW7 2AZ, UK; Department of Life Sciences, Imperial College London, Silwood Park Campus, Ascot, Berkshire, SL5 7PY, UK; Department of Biology, University of Oxford, 11a Mansfield Road, Oxford, OX1 3SZ, UK; Instituto de Biociências, Universidade Federal de Mato Grosso do Sul, Avenida Costa e Silva s/n Bairro Universitário, Campo Grande, Mato Grosso do Sul CEP 79070-900, Brazil; Institute of Evolution, University of Haifa, 199 Aba Khoushy Ave, Mount Carmel, Haifa, 3498838, Israel; Comparative Fungal Biology, Royal Botanic Gardens, Kew, Jodrell Laboratory, Richmond, TW9 3DS, UK; Department of Life Sciences, Imperial College London, Silwood Park Campus, Ascot, Berkshire, SL5 7PY, UK; Department of Biology, University of Oxford, 11a Mansfield Road, Oxford, OX1 3SZ, UK; Comparative Fungal Biology, Royal Botanic Gardens, Kew, Jodrell Laboratory, Richmond, TW9 3DS, UK

**Keywords:** anthraquinone, lichenized fungi, *Lecanoromycetes*, ABC-transporter, biosynthetic gene cluster, fungal evolution

## Abstract

Fungi produce a vast number of secondary metabolites that shape their interactions with other organisms and the environment. Characterizing the genes underpinning metabolite synthesis is therefore key to understanding fungal evolution and adaptation. Lichenized fungi represent almost one-third of *Ascomycota* diversity and boast impressive secondary metabolites repertoires. However, most lichen biosynthetic genes have not been linked to their metabolite products. Here we used metagenomic sequencing to survey gene families associated with production of anthraquinones, UV-protectant secondary metabolites present in various fungi, but especially abundant in a diverse order of lichens, the *Teloschistales* (class *Lecanoromycetes*, phylum *Ascomycota*). We successfully assembled 24 new, high-quality lichenized-fungal genomes de novo and combined them with publicly available *Lecanoromycetes* genomes from taxa with diverse secondary chemistry to produce a whole-genome tree. Secondary metabolite biosynthetic gene cluster (BGC) analysis showed that whilst lichen BGCs are numerous and highly dissimilar, core enzyme genes are generally conserved across taxa. This suggests metabolite diversification occurs via re-shuffling existing enzyme genes with novel accessory genes rather than BGC gains/losses or de novo gene evolution. We identified putative anthraquinone BGCs in our lichen dataset that appear homologous to anthraquinone clusters from non-lichenized fungi, suggesting these genes were present in the common ancestor of the subphylum *Pezizomycotina*. Finally, we identified unique transporter genes in *Teloschistales* anthraquinone BGCs that may explain why these metabolites are so abundant and ubiquitous in these lichens. Our results support the importance of metagenomics for understanding the secondary metabolism of non-model fungi such as lichens.

**Significance**

Lichen-forming fungi produce an immense diversity of secondary metabolite compounds, many of which have potentially useful bioactive properties. Most of the gene clusters responsible for compound production have not been characterized, especially in certain lichen lineages. In this study, we use comparative metagenomics to characterize putative secondary metabolite gene clusters for UV-protectant anthraquinone pigments in the *Teloschistales* clade. These clusters have the same core enzyme genes as many other lichen gene clusters but contain unique accessory genes that may have allowed anthraquinones to become the dominant metabolite group produced by this lineage. Our results suggest that shuffling gene cluster elements has led to metabolite novelty in the *Teloschistales*.

## Introduction

Fungi produce a high diversity of secondary metabolites, with functions including toxins (Rohlfs 2015; Boddy and Hiscox 2017), virulence factors (Williams et al. 2011; Boyce et al. 2019), and protection against stressful environments (Bills and Gloer 2017). Many of these compounds are used by humans as powerful bioactive molecules. Understanding the genetic basis for metabolite production and how evolution acts on those genes to generate chemical diversity is an important step for both understanding fungal diversity and adaptation and applications of biomolecules.

In fungi, genes involved in the same secondary metabolic pathway often cluster together on the chromosome (Naranjo-Ortiz and Gabaldón 2020). Gene clusters offer a series of evolutionary advantages over nonclustered genes, including tighter control of expression (Hurst et al. 2002), reduced likelihood of recombination disrupting interacting loci (Rokas et al. 2018), and prevention of accumulation of self-toxic products through the inclusion of genes encoding metabolite transporters within the cluster (McGary et al. 2013). Investigating variation in gene cluster content and structure across different taxa may therefore help us to understand the diversity and distribution of fungal secondary metabolites and the evolutionary processes that led to their formation (Slot and Gluck-Thaler 2019).

The shift from sequencing individual genes to whole genomes has allowed us to study the full BGC repertoire of a fungus rather than just a few of its core biosynthetic genes. Analyses have shown that BGCs are numerous, largely species-specific (reflecting variability in species chemistries), and importantly represent hotspots of evolution within the genome (Wisecaver et al. 2014; Koczyk et al. 2015; Theobald et al. 2018; Vesth et al. 2018; Gluck-Thaler et al. 2020). Lind et al. (2017) identified five key genetic processes that could explain BGC variation in fungi. These cover polymorphism in BGC functionality, polymorphism in position in the genome, polymorphism in allelic content, gains/losses of individual genes, and gains/losses of whole BGCs. Comparing the increasing number of fungal whole-genome sequences provides a way to test the contribution of these processes within a phylogenetic context. Major groups of fungi remain, however, relatively under-explored.

With approximately 19,000 currently named species, lichen-forming fungal symbionts represent almost one-third of the known *Ascomycota* (Lücking et al. 2017) and boast an impressive repertoire of secondary metabolite compounds (>1,000 compounds described to date; Huneck et al. 1996; Stocker-Wörgötter 2008; Elix 2014), including molecules that allow them to inhabit harsh environments like exposed rock surfaces. Lichens have historically been under-represented in studies of BGC diversity and evolution, however, due to challenges with growing axenic cultures for genome sequencing. In recent years, the application of metagenomic approaches has greatly increased the number of available lichen-forming fungal genomes and metagenome-assembled genomes (MAGs) of lichen mycobionts now equal or even exceed the quality of those obtained from pure cultured material (Tavares et al. 2014; Meiser et al. 2017; McKenzie et al. 2020). Furthermore, thanks to long-read sequencing, we are beginning to see the first reference-quality lichen mycobiont assemblies (McKenzie et al. 2020; Wilken et al. 2020; Allen et al. 2021; Singh, Armaleo, et al. 2021; Gerasimova et al. 2022). Nevertheless, lichenized-fungal genomes are still under-represented in public repositories, with only 107 full genome assemblies available for the *Lecanoromycetes* (the largest class of lichenized fungi containing almost 80% of described lichen species; Lücking et al. 2017), compared with 1,711 and 3,600 genomes available for the *Dothideomycetes* and *Sordariomycetes* classes, respectively, despite all three classes having similar numbers of described species (calculated using pipeline of Hill et al. 2021). Comparative analysis of lichen mycobiont genomes has shown that BGCs are as numerous as in other classes of the *Ascomycota*, if not more so (Armaleo et al. 2011; Bertrand et al. 2018a; Calchera et al. 2019; Pizarro et al. 2020; Gerasimova et al. 2022). Additionally, these studies consistently show that for a given lichen species the number of BGCs in the genome greatly outnumbers the known metabolites, suggesting that many secondary metabolites remain undiscovered.

A key challenge for genomic prospecting is linking genes or gene clusters to their metabolite products. In lichens, direct confirmation of gene function is hard because of their extremely slow growth, the complexity of maintaining them in axenic culture, and difficulties with establishing effective genetic manipulation techniques. Instead, most studies adopt a homology-based approach whereby lichen genes of unknown function are mapped to genes that have been experimentally characterized in model fungi (Bertrand et al. 2018b). This approach has been used to suggest putative genes for the lichen compounds atranorin (Gerasimova et al. 2022), olivetoric, and physodic acids (Singh, Armaleo, et al. 2021), gyrophoric acid (Singh et al. 2022), amongst others. One to one mapping is not always possible because of extensive duplication in lichen metabolite genes (Schmitt et al. 2005; Opanowicz et al. 2006; Muggia et al. 2008), production of multiple compounds of the same class, or production of different metabolites by regulation of a single BGC (Singh, Armaleo, et al. 2021; Singh et al. 2022). Nonetheless, genome analyses provide a starting point for predicting genetic repertoires, which in two cases have been confirmed by subsequent heterologous expression in non-lichen hosts (Kealey et al. 2021; Kim et al. 2021).

Here, we use a comparative metagenomic survey approach to search for gene clusters associated with production of a key lichen metabolite group, anthraquinones, in the order *Teloschistales* Zahlbr (*Lecanoromycetes*, *Ascomycota*). With over 1,000 species, the *Teloschistaceae* Zahlbr., the largest family in the order, is one of the most speciose lichen families (Arup et al. 2013; Lücking et al. 2017). During the Late Cretaceous, habitat switches from shaded epiphytic habitats to exposed rocky ones allowed this family to radiate into arid ecosystems across the globe, which is where we currently find their biodiversity hotspots (Gaya et al. 2015). This habitat switch coincided with secondary metabolite innovation at the onset of this family in the form of sun-screening anthraquinone production (Gaya et al. 2015). Anthraquinone evolution, therefore, appears interlinked with the evolutionary success of the *Teloschistales* lineage.

Anthraquinones are photoprotective pigments that are deposited in the upper layers (cortex) of the lichen and have been shown to efficiently absorb UV and blue light (Solhaug and Gauslaa 1996; Gauslaa and Ustvedt 2003). They are products of iterative multidomain polyketide synthases (PKS) enzymes (Type I PKS/PKSI). These enzymes are often characterized by their thioesterase (TE) domain structure, the domain responsible for cleaving the polyketide from the PKS (Li et al. 2010). In the case of fungal anthraquinones, their corresponding gene clusters consistently show a PKS that lacks a TE-domain adjacent to a distinct metallo-β-lactamase-type thioesterase (MβL-TE) gene (Awakawa et al. 2009; Chiang et al. 2010; Lim et al. 2012; Griffiths et al. 2016; Neubauer et al. 2016; Throckmorton et al. 2016). BGCs for different anthraquinones in nonlichenized fungi show a high level of sequence similarity and synteny suggesting anthraquinone production in these fungi is homologous. Whether this extends to lichenized fungi that produce much higher levels of anthraquinones has yet to be tested. Only two clusters have been putatively linked to anthraquinone biosynthesis in lichens so far (Wang et al. 2014; Bertrand et al. 2018b). Although these show apparent homology to non-lichenized fungi, comparisons of more species are needed to determine homology and explore anthraquinone BGC diversity across all lichen groups. A comparative approach will allow us to investigate how anthraquinone production transitioned from the relatively rare, minor lichen metabolite trait seen in most lineages into the dominant secondary metabolite and a near ubiquitous trait in the *Teloschistales*.

We still know very little about the BGCs underpinning anthraquinone biosynthesis in lichens, particularly in the *Teloschistales*, for which only three full genome sequences are publicly available. In this investigation, we increased the number of available lichen genomes by implementing a metagenomics approach to sequence and assemble 24 new lichen genomes, with a special emphasis on the *Teloschistales*. These genomes were then used alongside 21 published *Lecanoromycetes* genomes to perform a comparative genomic analysis of lichen BGCs to predict links between these clusters and key chemical compounds. We asked whether lichens present anthraquinone BGCs homologous to those of non-lichenized fungi, whether *Teloschistales* BGCs contain unique elements that could explain anthraquinone prevalence in this clade, and whether metabolite diversification in lichens occurs through gains and losses of entire BGCs or shuffling of components within BGCs. Using anthraquinones as a case study, we explore how fungal secondary metabolite innovation occurs and hypothesize a genetic mechanism for how lichenized clades such as the *Teloschistales* specialize to produce such high concentrations of compounds.

## Results

### Metagenomics Produces New High-Quality Mycobiont Genomes

We present 24 newly sequenced and de novo assembled *Lecanoromycetes* mycobiont genomes from metagenomic data, covering four families in three orders: the *Letrouitiaceae* and *Teloschistaceae* (*Teloschistales*), the *Umbilicariaceae* (Umbilicariales), and the *Graphidaceae* (*Ostropales*). Due to the high level of taxonomic uncertainty and ongoing revision of *Teloschistaceae* genus names, we opted for a conservative approach and used only established names as in Arup et al. (2013) (e.g., *Teloschistes* over *Niorma*). The metagenome-assembler MEGAHIT (Li et al. 2015) consistently produced the most complete (BUSCO %) and contiguous (N50) assemblies compared with MetaSPAdes (Nurk et al. 2017), which only produced higher quality assemblies in a handful of cases (taxa labeled with *^S^* in table 1).

**Table 1:** Genome Assembly Statistics for the Assembled *Lecanoromycetes* Mycobiont Genomes

Taxon	Assembly Size (Mbp)	No. Scaffolds	N50 (K)	BUSCO (%)	Duplicated BUSCO (%)	Predicted Genes	GenBank Accession
*Caloplaca aegea*	31.48	6,484	9	92.44	0.05	7,543	JALAIP000000000
*Caloplaca aetnensis*	31.90	2,773	24	95.72	0.06	9,101	JALAIC000000000
*Caloplaca ligustica*	24.20	1,915	19	89.27	0.23	7,863	JALAIE000000000
*Diploschistes diacapsis*	29.15	4,361	15	91.38	0.00	8,138	JALAIB000000000
*Flavoplaca oasis*	33.60	3,025	28	95.13	0.17	9,027	JALAIS000000000
*Gyalolechia ehrenbergii ^S^*	39.67	317	209	97.25	0.00	8,095	JALAIJ000000000
*Letrouitia leprolyta ^S^*	27.85	1,220	34	94.37	0.11	9,953	JALAIH000000000
*Letrouitia transgressa* 1 *^S^*	26.34	337	290	95.90	0.29	8,199	JALAIO000000000
*Letrouitia transgressa* (2)	27.99	501	211	96.37	0.23	8,581	JALAIG000000000
*Seirophora lacunosa* 1 *^S^*	38.78	428	146	97.89	0.06	8,161	JALAHX000000000
*Seirophora lacunosa* (2)	35.69	2,533	21	94.49	0.06	7,847	JALAIF000000000
*Seirophora villosa ^S^*	36.72	2,163	28	93.73	0.11	7,507	JALAHY000000000
*Teloschistes chrysophthalmus*	27.03	776	106	97.95	0.35	8,751	JALAIL000000000
*Teloschistes flavicans*	25.62	1,520	25	94.49	0.06	8,438	JALAIQ000000000
*Teloschistes peruensis*	26.73	966	82	97.71	0.41	8,483	JALAHW000000000
*Umbilicaria vellea*	28.68	3,107	14	90.56	0.17	7,140	JALAID000000000
*Usnochroma carphinea ^S^*	36.98	2,385	30	97.36	0.23	8,370	JALAHV000000000
*Variospora aurantia*	24.80	5,327	8	89.92	0.06	7,864	JALAHZ000000000
*Xanthomendoza fulva*	29.80	952	129	97.83	0.29	8,822	JALAIN000000000
*Xanthoria aureola ^S^*	38.52	5,150	12	96.54	0.53	10,011	JALAII000000000
*Xanthoria mediterranea ^S^*	32.48	447	140	97.89	0.12	9,057	JALAIA000000000
*Xanthoria* sp. 1	54.66	8,581	11	90.68	1.14	11,606	JALAIM000000000
*Xanthoria* sp. 2	37.07	2,884	27	94.43	0.17	9,784	JALAIR000000000
*Xanthoria steineri ^S^*	30.64	2,267	35	97.71	0.23	9,436	JALAIK000000000

Note.—Taxa with superscript *^S^* highlight assemblies where MetaSPAdes performed best. Assembly size is indicated in Megabase pairs and N50 in thousands of bases.

We developed a new pipeline to isolate the mycobiont reads from the lichen metagenome using an iterative, combined approach of the Blobtools workflow (Laetsch and Blaxter 2017), UniRef and custom lichen database blast searches, and CONCOCT metagenome binning (Alneberg et al. 2014) (see supplementary fig. S1, Supplementary Material online, for an example of the pipeline output). We developed this pipeline as exploratory data analysis showed AT-rich regions that make up significant proportions of the mycobiont genome were not being assigned to the mycobiont using BLAST or database methods like Kraken (Wood and Salzberg 2014), an issue also observed by Targirdzhanova et al. (2021). The full pipeline with accompanying bioinformatics scripts is available here: https://github.com/theo-llewellyn/TeloschistalesMetagenomics. Mycobiont assembly sizes range from 25.18 Megabase pairs (Mbp) (*Caloplaca ligustica*) to 54.66 Mbp (*Xanthoria* sp. 1) with a mean assembly size of 32.47 Mbp. These values fall within the range of published *Lecanoromycetes* genome assemblies sequenced from cultured samples (supplementary fig. S2, Supplementary Material online). All assemblies were over 90% complete for BUSCO genes (mean 94.88%) except for *Caloplaca ligustica* (89.27%) and *Variospora aurantia* (89.92%). Duplicated BUSCO percentages were all below 0.6% (mean 0.21%) except for *Xanthoria* sp. 1 (1.14%). Completeness scores are comparable to, or higher than, published *Lecanoromycetes* genomes obtained from cultures. The number of predicted genes varied considerably ranging from 7,140 (*Umbilicaria vellea*) to 11,606 (*Xanthoria* sp. 1).

Assembly quality metrics were comparable to those from published genomes obtained from cultured mycobionts, yet with slightly more variation (supplementary fig. S2, Supplementary Material online). Metagenome assemblies were generally more fragmented than genomes sequenced from cultured samples (supplementary fig. S2, Supplementary Material online), an unsurprising result given that high molecular weight DNA is easier to extract from an actively growing culture. Assembly fragmentation does not appear to have affected gene annotation, with a similar number of predicted genes between culture-derived and metagenomic assemblies (supplementary fig. S2, Supplementary Material online).

**Fig. 1.**
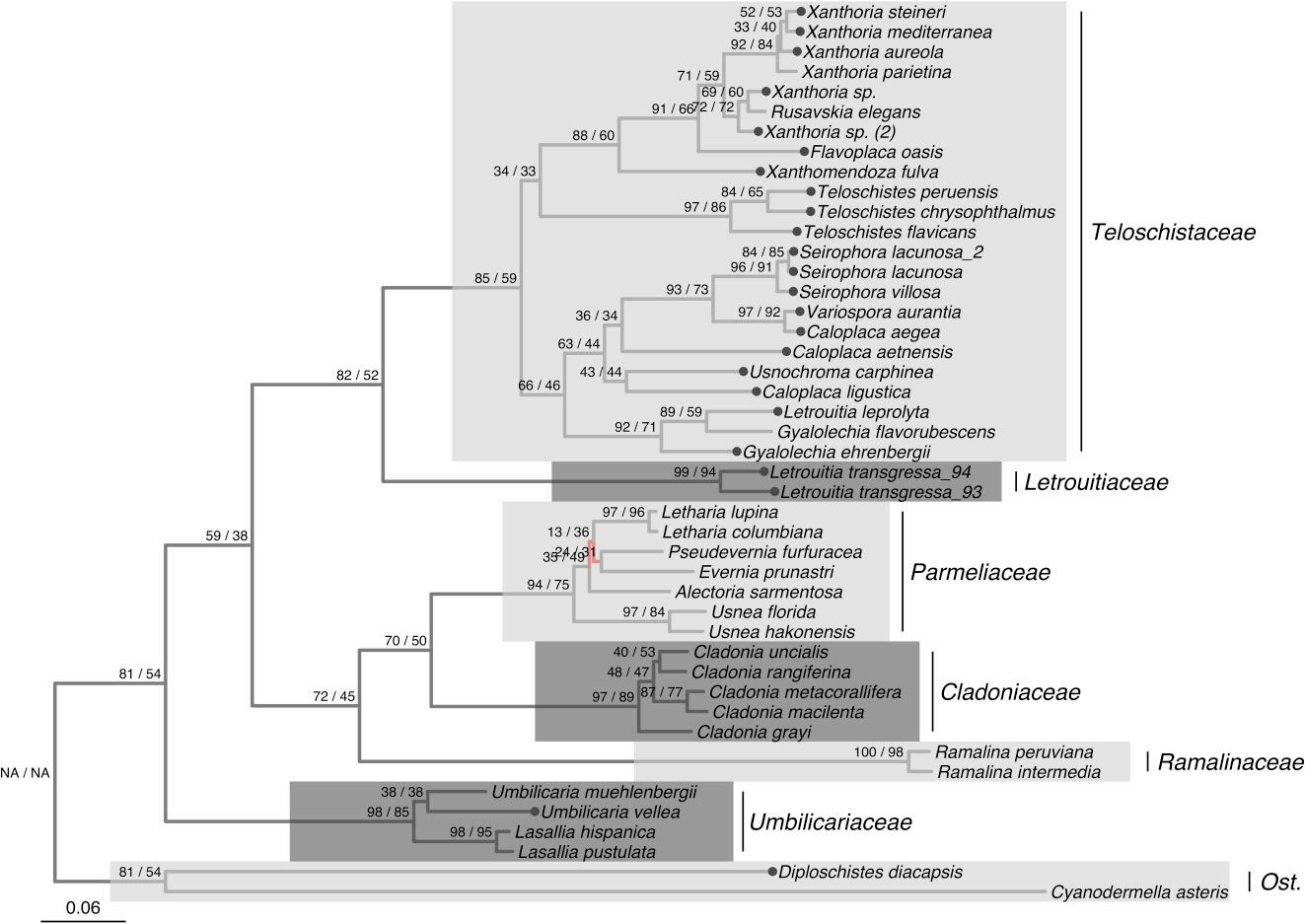
ML tree including representatives of the largest class of lichenized fungi, the *Lecanoromycetes* (*Ascomycota*), with special focus on the order *Teloschistales*, reconstructed with IQTree using a 2,214 single-copy gene combined dataset. All internodes, except for the one connecting *Umbilicaria muehlenbergii* and *U. vellea* and the red branches in the family *Parmeliaceae*, were significantly supported by both bootstrap and posterior probabilities. Red branches indicate a conflict with the ASTRAL tree. Support values before and after the forward slash indicate gene and site concordance factors, respectively. Dotted tips show taxa sequenced and assembled in this study. Families/order are labeled and highlighted by alternating white and gray blocks. Ost. = *Ostropales*.

We used both concatenation and coalescent-based approaches to reconstruct a whole-genome-scale phylogeny for our 24 newly sequenced mycobiont genomes, combined with a further 21 published *Lecanoromycetes* genomes. The final alignment consisted of 2,214 single-copy protein-coding genes. IQTree (Nguyen et al. 2015) and ASTRAL-III (Zhang et al. 2018) recovered largely the same topology except for two branches within the family *Parmeliaceae* (red branches in fig. 1). Differing branches showed high gene and site conflict, estimated using gene and site concordance factors (Minh et al. 2020). All internodes were significantly supported (UFBoot and LPP >95%) except for the monophyly of the genus *Umbilicaria* and the conflicting relationship within the *Parmeliaceae*. All families were recovered as monophyletic with >80% gene and >50% site concordance, suggesting low gene and site conflict. The three subfamilies identified in the *Teloschistaceae* (Gaya et al. 2012; Arup et al. 2013) were supported here, and all *Teloschistales* genera were monophyletic. The presence of a sample of *Letrouitia leprolyta* within *Gyalolechia* confirms a suspected erroneous microscopic identification and is likely to be described as a new taxon. Further study of this sample will be required.

### Lecanoromycetes Genomes Are Diverse in Secondary Metabolite Gene Clusters and Show High Dissimilarity

**Fig. 2.**
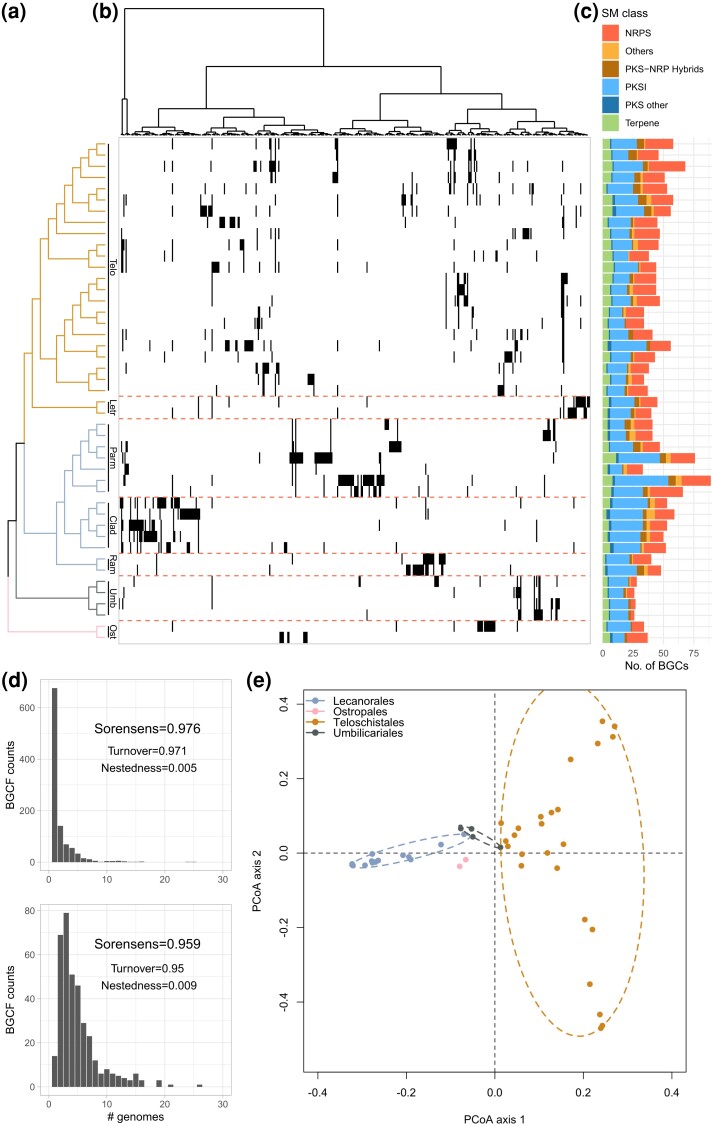
Diversity and distribution of secondary metabolite BGCs in the *Lecanoromycetes*. (*a*) Topology represents the ML tree shown in figure 1 with branches colored by taxonomic order as per figure 2*e* legend. (*b*) Presence–absence matrix of BGCFs in each genome (rows), showing only PKSI class BGCFs for readability. Each matrix column represents a single BGCF, with columns ordered by presence–absence similarity profiles across genomes. Dendrogram above matrix displays the BGCF similarity profile clustering produced with hclust function in R. Horizontal, orange dashes delineate taxonomic families. Presence = black, absence = white. (*c*) Stacked bar plot showing the number of predicted BGCs per genome. Colors represent different BGC classes. (*d*) Histograms showing the number of genomes per BGCF. The top histogram shows the BiG-SCAPE results using a strict cutoff of 0.46 to group BGCs into BCGFs and the bottom histogram showing the results using a more relaxed cutoff of 1. Beta diversity statistics at both cutoffs are shown on each graph and are separated into their turnover and nestedness components. (*e*) PCoA of all BGCF presence–absence profiles shown in (*b*). Colors represent taxonomic orders with ellipses drawn around orders with three or more points.

Annotation of biosynthetic gene clusters (BGCs) in the 45 *Lecanoromycetes* genomes (24 de novo assemblies and 21 previously published) identified a total of 2,073 BGCs. The average number of BGCs per genome was 46 ± 12.80. In a fragmented assembly, large BGCs may be split across multiple contigs which could lead to them being erroneously detected as two or more separate clusters. We used a linear model implemented in R to test whether assembly fragmentation (N50) affected number of BGCs detected per genome. A partial *F*-test showed no significant effect of assembly fragmentation (N50) on number of detected BGCs (supplementary fig. S3, Supplementary Material online). Therefore, it does not appear that we overestimated the number of BGCs per genome in this study. Type 1 polyketide synthases (PKSI) and nonribosomal peptide synthases (NRPS) were the most abundant BGC classes across all genomes (fig. 2*c*). BiG-SCAPE network analysis (Navarro-Muñoz et al. 2020) grouped the BGCs into 1,033 biosynthetic gene cluster families (BGCFs) using pairwise distances calculated from similarity of BGC protein domain content, sequence identity, domain order and copy number. We included reference BGCs with known metabolite products from the MIBiG database (Medema et al. 2015) to infer putative BGC metabolite products in our lichen BGCs. Of the 1,033 BGCFs, 22 contained MIBiG reference BGCs (table 2). The largest BGCF was an NRPS family containing 25 BGCs. The majority of the 22 families containing MIBiG reference BGCs were PKSIs.

**Table 2:** Results of BiG-SCAPE Clustering of BGCs into Families

BGC Class	No. Families	Average No. BGC/Family	Largest Family (No. BGCs)	Families w/MIBiG BGC
PKSI	421	2	16	16
NRPS	361	2	25	0
PKS-NRP Hybrid	73	2	13	4
PKS other	21	3	16	1
Terpene	99	2	24	1
Others	58	2	5	0
Total	1,033	2	NA	22

BGCFs mostly contained BGCs present in one or two taxa/genomes (fig. 2*d*, top plot), suggesting that BGCs are highly dissimilar between taxa. We estimated BGC content turnover between genomes using beta diversity, which in this context represents the proportion of BGC dissimilarity due to each genome containing a unique set of BGCs rather than a case of nestedness where genomes with fewer BGCs are simply subsets of more diverse genomes. Beta diversity (Sorensen's) showed BGC content was highly dissimilar between genomes and this was due mainly to turnover (Sorensen's beta diversity = 0.976: turnover = 0.971, nestedness = 0.005; Baselga 2010), suggesting each genome contains a largely unique set of BGCs. To test whether the high dissimilarity of BGCs was due to BiG-SCAPE being too strict when grouping BGCs into families, we performed the same analysis at the most relaxed cutoff value the algorithm permitted. The relaxed cutoff results showed a lower proportion of singleton BGCFs; however, the overall pattern remained, with the majority of BGCFs being represented by less than five taxa (fig. 2*d*, bottom). Beta diversity decreased slightly to 0.959, with the majority still due to turnover (turnover = 0.95, nestedness = 0.009).

Some blocks of BGCFs in figure 2*b* (showing only PKSI class for readability) appear to show phylogenetic patterns to their distributions. A principal coordinates analysis (PCoA) of BGCF profile dissimilarity (measured via Jaccard dissimilarity indices) suggests some heritability in BGCF profiles, as coloring the points by taxonomic order reveals four clusters representing the orders *Lecanorales*, *Ostropales, Teloschistales*, and *Umbilicariales* (fig. 2*e*), with little overlap between the clusters. A Mantel test showed a significant correlation between the phylogenetic distance of taxa and their BGCF profile dissimilarity as measured via Jaccard dissimilarity indices (Mantel statistic R: 0.6696, *P-value* = 0.001). PERMANOVA analysis further supported this, showing phylogenetic signal significantly explained variation in BGCF profile dissimilarity albeit weakly (phylogenetic principal component 1: *R*^2^ = 0.06998, *P-value* = 0.0001, phylogenetic principal component 2: *R*^2^ = 0.03987, *P-value* = 0.0001).

### Gene Cluster Family Analysis Identifies Putative Anthraquinone Clusters in Lecanoromycetes Genomes

**Fig. 3.**
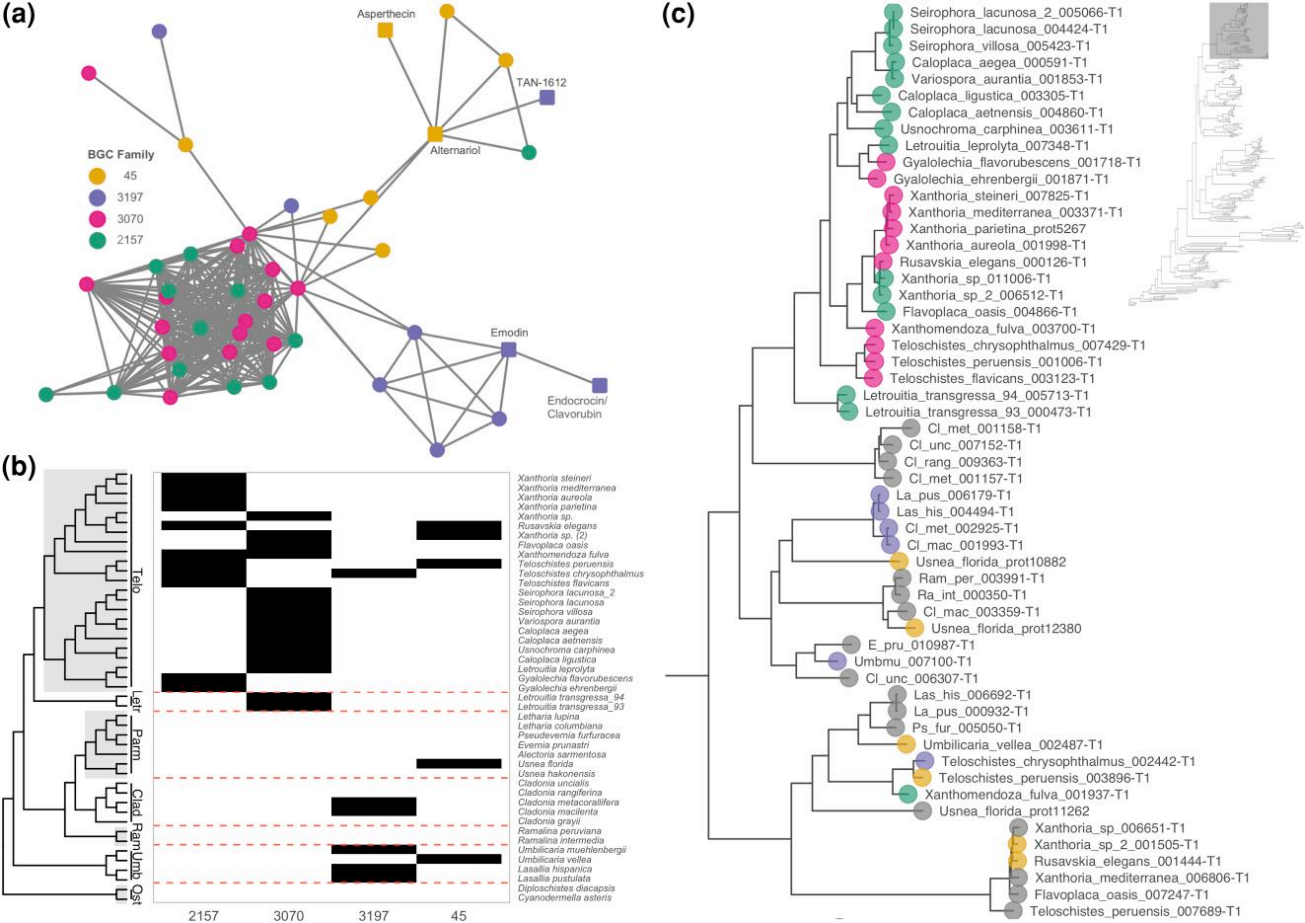
Putative anthraquinone BGCFs. (*a*) BiG-SCAPE similarity network linking BGCs (nodes) that showed a pairwise similarity score below the 0.46 cutoff established in this study. Node color shows to which BGCF each BGC was assigned by affinity clustering. Square nodes indicate MIBiG reference gene clusters that have been experimentally linked to a compound via genetic manipulation. (*b*) Presence–absence matrix of the four putative anthraquinone BGCFs plotted against the phylogenomic tree shown in figure 1. Orange dashed lines delineate lichen taxonomic families. (*c*) Zoomed in clade of the ML gene tree for the core PKS genes including the BGCFs shown in A and B. Colored dots at tips show which BGCF the 39 core PKS genes belong to, gray dots are sequences that do not belong to the four putative anthraquinone BGCFs. Full tree including all 340 sequences found for this orthogroup is depicted in the inset, where the clade of interest is highlighted with a gray rectangle.

To identify putative anthraquinone BGCs, we restricted our search to PKSI BGCFs given that anthraquinones are aromatic polyketides. We searched for families containing reference MIBiG clusters linked to anthraquinones or structurally similar compounds. Four such families (BGCFs 45, 3,197, 3,070 and 2,157) were found containing 39 *Lecanoromycetes* BGCs linked to following the MIBiG clusters: the anthraquinones emodin in *Escovopsis weberi*, asperthecin in *Aspergillus nidulans* FGSC A4 and endocrocin/clavorubin in *Claviceps purpurea*, and the structurally related alternariol in *Aspergillus nidulans* FGSC A4 and TAN-1612 *Aspergillus niger*. The BiG-SCAPE similarity network connected all four BGCFs into a single network, further suggesting that gene clusters within these BGCFs are involved in the biosynthesis of similar compounds (fig. 3*a*). The distribution of these four BGCFs across the *Lecanoromycetes* genome-scale phylogeny is shown in figure 3*b*. All four BGCFs are present in the *Teloschistales* clade and all *Teloschistales* genomes we studied contain a gene cluster in at least one of the four BGCFs. BGCF 3,197 is also found in the *Umbilicariaceae* and *Cladoniaceae* and BGCF 45 also in the *Umbilicariaceae* and *Parmeliaceae*.

We then extracted the core PKS enzyme amino acid sequences from the 39 *Lecanoromycetes* BGCs belonging to these four BGCFs. All 39 PKS amino acid sequences belong to a single group of homologous genes (orthogroup), containing a further 301 sequences that were not present in our four putative anthraquinone BGCFs. The phylogenetic gene tree of this orthogroup is shown in figure 3*c* (amino acids were aligned and filtered using MAFFT and GUIDANCE2 and tree reconstructed using RAxML-ng, see methods for full details). The putative anthraquinone PKS sequences fall within a narrower supported monophyletic group within the gene tree (gray box in inset and zoomed in clade in fig. 3*c*) that contains an additional 17 sequences indicated with gray dots at tips. Therefore, the core PKS genes in our putative anthraquinone BGCs are homologous to many other PKS genes, including PKSs from lichens that are not known to produce anthraquinones.

### Anthraquinone BGCs in the *Teloschistales* Clade Have a Conserved Four-Gene Structure Including a Unique ABC-Transporter

Functional annotation with PANTHER suggested three potential functions for the PKS sequences within our putative anthraquinone PKS orthogroup (supplementary fig. S4*a*, Supplementary Material online). When plotting these functions on the orthogroup gene tree (supplementary fig. S4*b*, Supplementary Material online), a distinction is visible with most proteins being annotated as “conidial pigment polyketide synthase ALB1” proteins, whilst our putative anthraquinone PKSs (shown in fig. 3*c*) were annotated as either “atrochrysone carboxylic acid synthase-related” or “APTA-related.” Atrochrysone carboxylic acid is a common intermediate in anthraquinone biosynthesis in non-lichenized ascomycetes and AptA is the *Aspergillus nidulans* gene involved in the production of the anthraquinone asperthecin.

We followed PANTHER analysis with protein domain analysis of our putative anthraquinone PKS amino acid sequences. This identified four of the five key Pfam domains expected within a nonreducing PKS protein: SAT-KS-AT-ACP. Although the canonical product template (PT) domain, the fifth key PKS domain, was not detected using Pfam, subsequent BLAST analysis showed it to be present in all *Teloschistales* anthraquinone PKSs. Pfam also failed to annotate the PT domain of the MIBIG anthraquinone BGCs that were linked to our putative anthraquinone BGCs. This suggests the PT domains in fungal anthraquinone clusters may contain mutations that prevent them being annotated as such by Pfam.

**Fig. 4.**
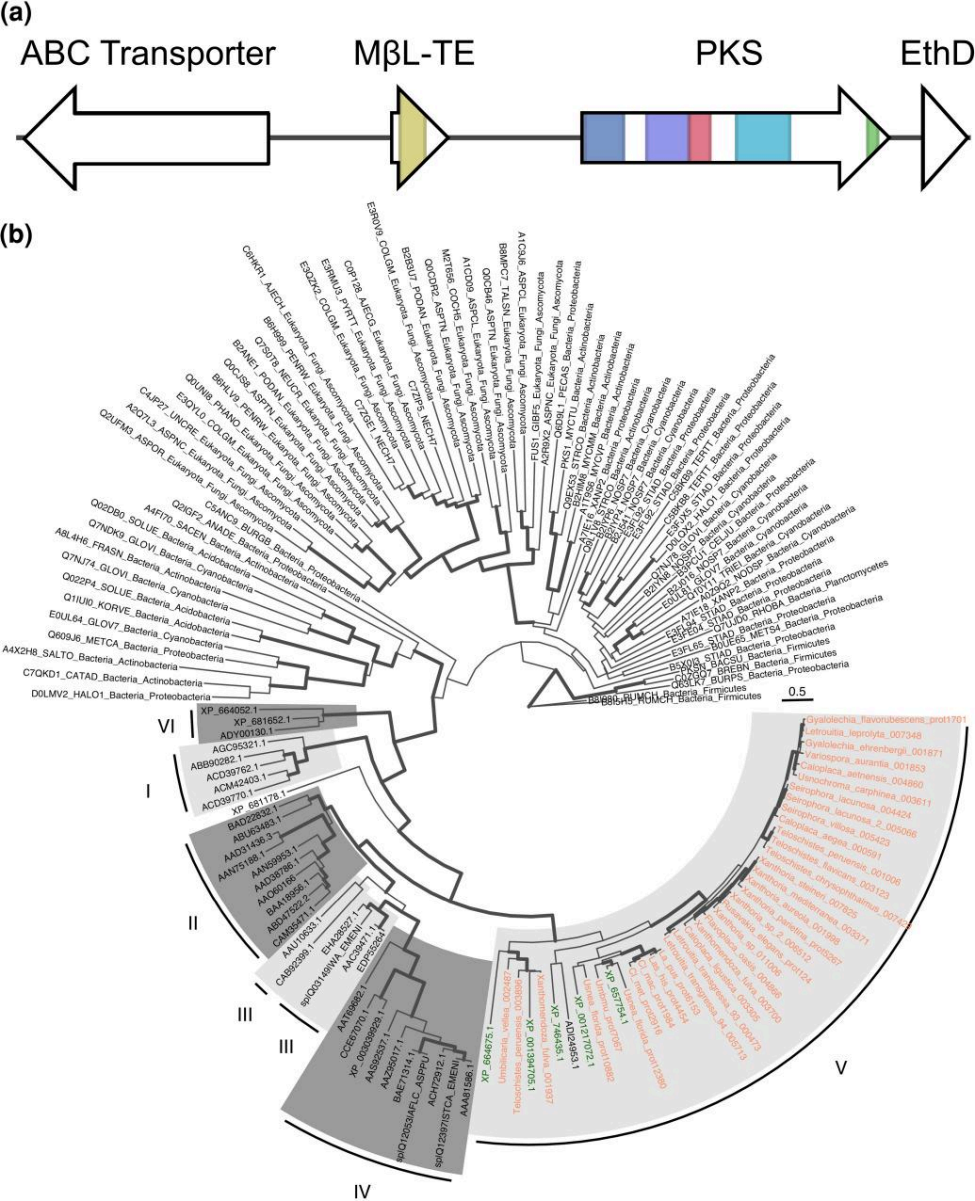
(*a*) BGC showing the four common genes in *Teloschistales* putative anthraquinone BGCs. From left to right: ABC-transporter, MβL-TE, PKS (with SAT-KS-AT-ACP domains), and gene containing EthD domain. BGC shown is from the *Variospora aurantia* genome. (*b*) ML phylogeny reconstructed using the PT domain (Pfam: PF14765) and a HMM alignment. Bold branches indicate bootstrap support ≥95%. Alternating dark and light grey blocks highlight the PT classification of Liu et al. (2015). All lichenized-fungal putative anthraquinone sequences from this study fall within group V. Putative *Lecanoromycetes* anthraquinone sequences are those with orange text and sequence ADI24953.1. Green text signifies sequences from genes experimentally confirmed to produce anthraquinones.

In the putative anthraquinone BGCs belonging to *Teloschistales* taxa, besides the core PKS gene, three additional genes were consistently and uniquely present (fig. 4*a*). The first gene encodes an ATP-Binding Casette (ABC) protein, which is a transmembrane efflux pump commonly found in fungal cells that actively expel metabolites. The second gene was annotated as encoding a MβL-TE. TEs cleave the final polyketide from the PKS enzyme and can exist either as a domain within a PKS protein or as a separate protein. The third additional gene was annotated as encoding a dehydratase with a single EthylD domain. For *Usnochroma carphinea*, this domain was instead found within the PKS gene. The order of these four genes was the same, when present, across all our putative anthraquinone gene clusters in the *Teloschistales*, although some clusters contained additional genes. Some *Teloschistales* genomes showed exceptions to this four-gene core structure, however. In addition to the aforementioned *Usnochroma carphinea* variation, *Seirophora villosa* was missing the EthD gene, *Xanthoria* sp. 1 was missing the ABC-transporter, and *Caloplaca aegaea* only had the core PKS gene. Whilst non-*Teloschistales* BGCs in our putative anthraquinone group all contained the PKS gene and some also contained the EthD and/or MβL-TE genes, the ABC-transporter gene was exclusive to *Teloschistales* BGCs.

### Phylogenetics Shows Putative Anthraquinone PKSs From Lichenized Ascomycetes Are Highly Syntenic With Those From Non-lichenized Ascomycetes

Given that the PT domain is indicative of compound structure and has been used to classify fungal PKSs, we extracted the PT domains from our 39 anthraquinone PKSs and combined them with 44 additional PT domains from fungal NRPKSs with known metabolite products (curated by Liu et al. 2015) for phylogenetic analysis. We aligned these PT domains with a further 76 bacterial and fungal PT domains from the Pfam seed alignment (PF14765) using a hidden Markov model (HMM) approach (see Methods). Eight sequences did not pass the filtering/quality control steps of the script leading to a final alignment of 151 sequences.

In the reconstructed maximum-likelihood (ML) tree, our putative anthraquinone PT domains formed a significantly supported grouping with the Liu et al. (2015) fungal PT domains with known products (fig. 4*b*). Within this clade, five supported subgroups (posterior probability >0.95) are highlighted in figure 4*b*. As these subgroups match exactly to five of the eight groups identified by Liu et al. (2015), we gave them the same numbering used in Liu et al. (2015). Our putative anthraquinone PT domains (orange names at tips in fig. 4*b*) fell within group V along with sequences from nonlichenized-fungal PKSs responsible for the anthraquinones endocrocin, atrochrysone/emodin and atrochrysone carboxylic acid/monodictyphenone, and the anthraquinone-related compounds alternariol and TAN-1612 (green tip names in fig. 4*b*). Within group V all *Teloschistales* anthraquinone PT domains form a single supported group. *Teloschistes peruensis* and *Xanthomendoza fulva* have an additional copy of the PKS gene (sequences Teloschistes_peruensis_003896 and Xanthomendoza_fulva_001937 in fig. 4*b*), which falls outside this supported group but still within group V.

## Discussion

This study presents 24 new lichenized-fungal genomes, focusing on the diverse and under-sampled *Teloschistales* order. Our metagenomic approach successfully isolated mycobiont genomes from lichen metagenomes, producing complete assemblies with good contiguity. A whole-genome phylogeny including our new genomes combined with 21 other genomes (20 lichenized and one non-lichenized fungus) of the *Lecanoromycetes* class shows high support and low conflict for all the families and all but one genus included. Secondary metabolite BGCs are numerous in the studied lichen genomes and a comparative analysis shows the BGC repertoire is highly dissimilar among taxa. Comparative genomics revealed a set of BGCs potentially responsible for anthraquinone biosynthesis. Functional annotation of these clusters supported their involvement in anthraquinone metabolism and uncovered a conserved four-gene BGC structure we believe to be shared uniquely across the *Teloschistales*. Further phylogenetic analysis of our anthraquinone PKS PT domains, a key domain in classifying fungal PKS function, showed PT domains were highly similar to those of anthraquinone PKSs from non-lichenized fungi, providing additional support for them producing anthraquinones.

### Metagenomics Is a Reliable Alternative to Axenic Cultures But Still Requires Manual Data Curation

MAGs are now commonplace in the field of lichen genomics and have consistently been demonstrated as reliable alternatives to culture-derived genomes (Tavares et al. 2014; Meiser et al. 2017; McKenzie et al. 2020; Singh, Armaleo, et al. 2021; Singh et al. 2022). The bioinformatic analysis of lichen metagenomes still presents challenges, however. For example, in this study, the mycobiont filtering step, though effective, requires manual curation when deciding which metagenome bins are to be merged. Tools such as metaWRAP (Uritskiy et al. 2018) can automate this process, but are currently restricted to prokaryote datasets (Parks et al. 2015). Increasingly, scientists are recognizing the importance of eukaryotes (especially *Fungi*) in microbial communities (West et al. 2018) and eukaryote-focused tools are being developed (Karin et al. 2020; Saary et al. 2020). Euk-CC (Saary et al. 2020) is one example that has recently been tested on a lichen metagenome (Tagirdzhanova et al. 2021), yet still required manual merging of bins to capture the AT-rich regions of the mycobiont genome. AT-rich regions have been frequently observed in non-lichenized fungal genomes, often constituting a significant proportion of the total genome size and containing genes with important adaptive roles (Rouxel et al. 2011; Testa et al. 2016). Based on our results, it seems these regions are also relevant for lichen mycobionts and should not be excluded during mycobiont filtering. Following Tagirdzhanova et al. (2021), we tested Euk-CC on our dataset but found that it failed to recover significant proportions of the mycobiont genomes. Therefore, we developed our own pipeline combining CONCOCT and BlobTools with UniRef and custom lichen database blast searches, which worked successfully on lichens across diverse lineages. As more lichen mycobiont genomes become available, we can use them as training data to develop lichen-specific metagenome binning tools and address this analytical bottleneck.

In addition, we still face shortcomings when it comes to assessing “completeness” of fungal genome assemblies using current bioinformatic tools. Assembly size can differ significantly from genome sizes estimated via experimental approaches such as cytometric analysis. Even highly complete BUSCO scores, which quantify the presence of a set of lineage-specific core genes, can be seen in genomes that are less than 50% complete based on cytometric data (Le Cam et al. 2019; Hill et al. 2021). In our case, flow cytometry would provide an important benchmark when assessing the effectiveness of our metagenomic bioinformatic pipeline to isolate complete mycobiont genomes. Unfortunately, cytometric data of lichenized fungi are still lacking (Hill et al. 2021), again due to difficulties with culturing lichen mycobionts. However, axenic cultures are not always necessary for cytometric analysis, as demonstrated by the work of (Tavares et al. 2014) on fungal plant pathogens.

Our results support the idea that metagenomics can be used to target under-represented lineages in the *Lecanoromycetes* such as the *Teloschistales*. Recently, Resl et al. (2022) implemented a similar approach to generate 18 MAGs covering diverse lineages and lifestyles of the *Lecanoromycetes*. Lichen MAGs can therefore be successfully incorporated into whole-genome-scale phylogenetic trees to investigate evolutionary and systematics-based hypotheses (Resl et al. 2022). The phylogenomic tree reconstructed in this study (fig. 1) agrees with other genome-scale trees of the *Ascomycota* (Shen et al. 2020; Li et al. 2021). Our tree is also largely in agreement with Pizarro et al.'s (2018) genome-scale tree focusing on the *Parmeliaceae* family, except for the placement of *Usnea*, which they recover as sister to the *Evernia*/*Pseudevernia* clade whereas we recover it as sister to the rest of the *Parmeliaceae* albeit with low gene and site concordance (Pizarro et al. 2018). At the order level, the phylogenomic tree shown in figure 1 matches that of the most comprehensively sampled and complete multigene phylogeny of the *Lecanoromycetes* (Miadlikowska et al. 2014).

### Lichen PKS Clusters Are Highly Dissimilar But Share Core PKS Genes

The increased number of sequenced fungal genomes allows for comparative analysis of BGCs amongst many species. Here we performed a comparative investigation of entire BGC profiles in lichens and the *Lecanoromycetes* class in particular. We observed high dissimilarity of BGC profiles between *Lecanoromycetes* genomes. This is not the first time that such high diversity of BGCs has been observed in fungi. In non-lichenized ascomycetes, similar patterns of dissimilarity have been observed at phylum (Robey et al. 2021), class (Gluck-Thaler et al. 2020), genus (Vesth et al. 2018), and species level (Lind et al. 2017). In lichens, BGC content can even vary between populations of the same species at different altitudes (Singh, Calchera, et al. 2021).

The absence of BGCs in certain groups may be explained by missing regions of the genome due to incomplete assemblies. However, we deem it unlikely as gene completeness scores were high across all analyzed genomes and the same pattern of high BGC dissimilarity has been observed in studies that incorporate long-read, reference-quality assemblies (e.g., Gluck-Thaler et al. 2020). Previous work has linked the high dissimilarity of fungal BGCs to speciation (Lind et al. 2017; Gluck-Thaler et al. 2020). Because the lichen clades in this study cover distant taxonomic groups and diverse ecological niches, speciation processes such as niche differentiation may well be contributing to the high dissimilarity we observe in the *Lecanoromycetes* BGC profiles, as shown by Singh, Calchera, et al. (2021) at the population level. Despite the high BGC dissimilarity, Mantel and PERMANOVA tests both identified significant effects of phylogenetic relatedness on BGC profile dissimilarity suggesting that more closely related taxa still have more similar BGC profiles.

We aimed to test two of the five sources of BGC variation identified by Lind et al. (2017); namely whether BGC diversity at metabolite class level occurs due to gains and losses of entire clusters, or more simply to shuffling/rearrangement of existing core genes with different tailoring and transport genes. Taking the case of our putative anthraquinone BGCs, we observe that the BGCs were mostly restricted to the *Teloschistales* and a handful of other taxa. However, when we extracted the core PKS gene and searched for homologues within the 45 lichen genomes studied, we found many orthologues and paralogues across all the genomes in the dataset, including taxa that do not produce anthraquinones. It therefore appears that the same shared ancestral PKS gene is used for different purposes in different lichen families. Certain paralogous PKS genes show widespread distribution across lichenized fungi (Opanowicz et al. 2006; Muggia et al. 2008), supporting this idea, and orthologous PKSs producing different compounds have been identified in both lichenized and non-lichenized fungi (Kroken et al. 2003; Koczyk et al. 2015; Singh, Armaleo, et al. 2021; Singh et al. 2022). This idea is further supported by the work of Gerasimova et al. (2022) which showed that all nonreducing PKS genes in a dataset of 23 lichen-forming fungal genomes belonged to a single orthogroup.

Our results therefore suggest that for *Teloschistales* anthraquinones, BGC diversification occurred via rearrangement of existing core genes with different accessory enzymes. To confirm this, we would need to repeat the networking analysis for the additional 301 PKS homologues (represented in fig. 3*c*, inset) and assess whether they form part of functional BGCs. If so, we could link them to their metabolite products with the same approach used in this study. There is still another possibility, however, that current orthologue identification approaches may be artificially grouping PKS genes that are structurally similar, but not truly orthologous. We believe this to be unlikely given the stringent filtering used when aligning the orthogroup amino acids and the high support seen in the corresponding gene tree.

### Putative Anthraquinone BGCs in the *Teloschistales* Contain Four Core Genes

Of the 25 *Teloschistales* genomes analyzed in this study, 22 contained a putative anthraquinone BGC consisting of four genes: a PKS gene, an EthD gene, a MβL-TE, and an ABC-transporter. *Seirophora villosa* and *Xanthoria* sp. 1 genomes were missing one of the four genes and the *Caloplaca aegae* genome only contained the core PKS gene. Whether these genes are truly missing or due to fragmented/partially assembled contigs will need to be explored in future work with additional data.

The presence of ABC-transporter genes within the BGC is noteworthy. ABC-transporters are transmembrane efflux pumps found across the tree of life that actively transport metabolites across cell membranes (Stergiopoulos et al. 2002). In fungi, they are used to expel antibiotics produced by hosts or competitors (Urban et al. 1999) and to pump out endogenously produced metabolites to prevent toxic accumulation (Amnuaykanjanasin and Daub 2009). Their ability to transport a broad spectrum of metabolites can also lead to fungal multidrug resistance, a pressing issue in public health (Andrade et al. 2000; Higgins 2007).

Anthraquinones are toxic metabolites clearly produced in large quantities by the lichen taxa in the order *Teloschistales*. Having ABC-transporters adjacent to anthraquinone PKSs could potentially allow for efficient transport of anthraquinones out of the cell before they cause damage to the mycobiont cells and the other symbiont partners. They would also explain how *Teloschistales* lichens are able to accumulate such large amounts of anthraquinone crystals in the thallus cortex and reproductive structures. Antitoxin ABC-transporters are commonly found within fungal secondary metabolite BGCs (Keller 2015) and have been shown to provide self-protection specifically against the product of the BGC in which they are found (Gardiner et al. 2005). ABC-transporters have been identified in the usnic acid BGC of *Evernia prunastri* (Pizarro et al. 2020) and two uncharacterized BGCs in *Cladonia uncialis*, one of which was putatively linked to production of the anthraquinone emodin (Bertrand et al. 2018b). The same *C. uncialis* genome was included in this study, but no putative anthraquinone BGCs were identified (fig. 3).

Efflux pumps can be costly, diverting energy away from growth and DNA repair (El Meouche and Dunlop 2018). In *Teloschistales* lichens this may be compensated by their extremely slow growth and the fact that lichen anthraquinones themselves are sun-screening pigments, thereby shielding DNA from UV radiation (Solhaug and Gauslaa 1996; Gauslaa and Ustvedt 2003). Within the BGCFs containing our putative anthraquinone BGCs, only the *Teloschistales* BGCs contained ABC-transporters. This may explain why these compounds are so abundant and widespread in this order of lichens. The non-*Teloschistales* genera *Lasallia*, *Umbilicaria*, *Cladonia*, and *Usnea* that are also found in these BGCFs have the core PKS machinery to produce anthraquinones but may be limited in synthesizing large quantities due to toxic buildup within the cells and a lack of transporters to expel them. These non-*Teloschistales* taxa may also be synthesizing anthraquinones as intermediates in the synthesis of other compounds (as is the case for monodictyphenone synthesis in non-lichenized fungi; Chiang et al. 2010; Neubauer et al. 2016) and so are converting anthraquinones into other, potentially less toxic compounds before toxic accumulation can occur. Exploring how these ABC-transporters are regulated in the *Teloschistales* would allow for a greater understanding of anthraquinone biosynthesis and how it may be affected by environmental or biological factors.

The observed coupling of PKSs with MβL-TEs instead of a TE domain appears to be a common feature of anthraquinone biosynthesis, first described for atrochrysone biosynthesis (Awakawa et al. 2009) and since identified for many other anthraquinones in nonlichenized *Pezizomycotina* (Chiang et al. 2010; Lim et al. 2012; Griffiths et al. 2016; Neubauer et al. 2016; Throckmorton et al. 2016). The phylogenetic analyses of fungal PKSs performed in this study also consistently group these PKSs together along with our putative anthraquinone PKSs (see group V of Li et al. 2010 and Liu et al. 2015; figure 4*b* here). Additionally, Betrand, Abdel-Hameed and Sorensen (2018b) observed this structure in the aforementioned emodin BGC from *C. uncialis* (Bertrand et al. 2018b). This high sequence similarity between anthraquinone PKSs of distantly related taxa both at the protein domain level and entire gene cluster level (see figs. 4*b*and 3*a* respectively), suggests that anthraquinone biosynthesis in the *Teloschistales* and other lichens is homologous to that of non-lichenized ascomycetes in the *Eurotiomycetes*, *Dothideomycetes*, and *Sordariomycetes* classes. This implies that anthraquinone biosynthesis could be a shared ancestral trait in the subphylum *Pezizomycotina*. A similar hypothesis was proposed for melanin biosynthesis in lichens, whereby certain lichen PKSs were homologous to melanin PKSs of nonlichen *Pezizomycotina* and shared a conserved structure of two ACP domains between the PT and TE domains (Pizarro et al. 2020). Ancestral-state reconstruction of anthraquinone type TE-less PKSs within the *Pezizomycotina* could explore this hypothesis further and investigate which factors control whether this trait is retained or lost. The fact that homologous anthraquinone PKSs are found between distantly related taxa within subphylum *Pezizomycotina* but not outside the subphylum, gives additional support to the theory that extensive PKS diversification occurred at the root of the *Pezizomycotina* clade via gene duplication and/or horizontal gene transfer (Kroken et al. 2003; Schmitt and Lumbsch 2009; Lawrence et al. 2011; Koczyk et al. 2015).

It is worth noting that an alternative strategy for anthraquinone production has been observed in the non-lichenized *Lecanoromycetes* species *Cyanodermella asteris*. The BGC responsible for producing the anthraquinone skyrin in this species does not contain the TE-less PKS/MβL-TE pairing and shows no apparent synteny with the lichen and nonlichen anthraquinone BGCs discussed in this study (Jahn et al. 2017). This highlights the importance of linking chemical phenotype to genotype as convergent evolution may lead to different genetic mechanisms producing the same or similar compounds.

Here we focused on the two largest and most diverse families (*Letrouitiaceae* and *Teloschistaceae*) of the four within *Teloschistales*, and the three subfamilies included in the largest family Teloschistaceae. The patterns we see are likely to apply to the whole order, however, given their diversity and chemical variability, an essential next step would require exploring anthraquinone BGC variation more extensively at the genus level in all families in the order, including the smaller *Brigantiaceae* and *Megalosporaceae* families. Special focus should be placed on *Megalosporaceae* and genera such as *Pyrenodesmia* given that these lineages appear to have secondarily lost anthraquinone pigments. Although these are difficult taxa to sample and sequence at the genome level, including them could allow us to investigate whether secondary pigment loss is due to entire BGC loss, as is the case for usnic acid in the lichen family *Parmeliaceae* (*Lecanorales*) (Pizarro et al. 2020), individual gene loss (e.g., loss of ABC-transporters) or another genetic mechanism.

## Conclusions

In this study, we implemented a metagenomics approach to assemble and analyze 24 new high-quality *Lecanoromycetes* genomes. Our comparative genomic analysis identified high diversity and dissimilarity in lichen BGCs with most gene clusters being species-specific. Using a similarity-based networking approach we identified a set of putative anthraquinone clusters in the *Teloschistales* with a conserved four-gene structure and high synteny to anthraquinone clusters from non-lichenized fungi. These clusters provide a starting point for future transcriptomic and genetic engineering studies to link gene clusters more definitively to specific anthraquinones and understand how pigment metabolism in lichens is controlled. Based on our analysis we believe the shuffling of pre-existing PKS genes with key accessory genes such as ABC-transporters may explain how *Teloschistales* lichens evolved the capacity to produce such high quantities of anthraquinones without suffering their cytotoxic effects.

## Materials and Methods

### DNA Extraction & Whole-Genome Sequencing

For newly sequenced lichen genomes, sampling was focused on the order *Teloschistales* (*Lecanoromycetes*, Ascomycota). We selected 22 samples covering 20 species to cover the three subfamilies included within the largest family *Teloschistaceae* and its sister family *Letrouitiaceae*. In addition, two non-*Teloschistales* taxa (*Diploschistes diacapsis*, *Umbilicaria vellea*) were sampled. Supplementary table S1, Supplementary Material online, includes locality and collection information for all samples. One sample of note is *Teloschistes peruensis* (family *Teloschistaceae*). The species was located along a small ridge crest (∼100 × 30 month) of sedimentary uplift superimposed with a powdery desert loess and cryptobiotic crust in the Lomas Amara in Peru. Another such habitat has yet to be found and the climatic, geological and topographic niche are clearly very unusual under today's conditions, possibly due to contraction of such climatic niche habitats and ecological degradation of other fog-oases (O. Whaley, personal communication). The core area of Lomas de Amara is incorporated and protected under national designation as an “Ecosistema Fragile” (Resolution No. 153-2018-MINAGRI-SERFOR) and *Teloschistes peruensis* has been assessed as critically endangered on the IUCN Red List (Ramos et al. 2021).

Lichen thalli that were clear from visible signs of other fungal contaminants were cleaned of any substrate debris, washed with double-distilled water, transferred to 2 ml Eppendorf tubes. These were ground to a fine powder using a Mixer Mill MM 400 (Retsch, Germany) and two sterile stainless-steel beads per tube. DNA was extracted using a modified CTAB protocol (Cubero and Crespo 2002) or alternatively the Qiagen DNeasy® Plant Mini Kit following the manufacturer's protocol (Qiagen, Redwood City, CA, United States). Extractions were quality checked using a Quantas^TM^ Fluorometer (Promega, UK) and NanoDrop™ 2000 Spectrophotometer (ThermoFisher Scientific, UK). Genome libraries were prepared using either the TruSeq Nano PCR-free DNA Kit (Illumina) or Nextera XT DNA Library Preparation Kit with a 550 bp insert size. Paired-end libraries (2 × 151 bp) were sequenced using a NovaSeq 6000 platform (Illumina, San Diego CA). Library prep and sequencing were performed at Macrogen (Macrogen Inc. South Korea).

### Metagenome Assembly and Mycobiont Genome Isolation

Illumina paired-end reads were quality checked using FASTQC v0.11.2 (Andrews et al. 2015). Low-quality bases and adapters were removed using Trimmomatic v0.36 (Bolger et al. 2014) with a four base sliding window and minimum Phred score of 15. Only reads with a mate pair post-trimming were used for assembly. Reads were assembled de novo into contigs using two assembly tools for comparison, MEGAHIT v1.2.9 (Li et al. 2015) and MetaSPAdes v3.15.1 (Nurk et al. 2017), both with default settings. Total assembly size, number of contigs >500 base pairs, and N50 were calculated using QUAST v5.0.2 (Gurevich et al. 2013). Genome completeness as measured by gene sets was quantified using BUSCO v4.0.2 (Manni et al. 2021) and the BUSCO Ascomycota lineage gene set—ascomycota_odb10.2019-11-20 (1,706 single-copy orthologs, https://busco.ezlab.org/list_of_lineages.html). QUAST and BUSCO metrics were used to choose the best of the two assemblies (i.e., MEGAHIT vs. MetaSPAdes) for subsequent steps.

Reads belonging to the *Lecanoromycetes* mycobiont were isolated from the metagenome using a new pipeline presented here consisting of a combination of the BlobTools v1.1.1 workflow (Laetsch and Blaxter 2017) and the metagenomic binning tool CONCOCT v1.1.0 (Alneberg et al. 2014). First, for BlobTools, read coverage was calculated using the function “bbmap” in BBTools (Bushnell 2014) and then converted to a BlobTools “covfile” using a one-line custom perl script (https://github.com/theo-llewellyn/TeloschistalesMetagenomics/blob/main/mycobiont_filtering/BlobTools/bbmap.sh). Metagenome contigs were then taxonomically identified using a double blast approach: 1) a DIAMOND BLASTx (Buchfink et al. 2014) against the Uniref90 database (Bateman 2019) and 2) a BLAST+ BLASTn (Camacho et al. 2009) against a custom database of all published genomes available in NCBI and JGI Mycocosm classified under *Lecanoromycetes*. Both blasts used an e-value cutoff of 1 × 10^−25^. BlobTools was then used to visualize the read coverage, GC-content, and taxonomic identification (phylum level) of all contigs. For the CONCOCT metagenome binning step, all metagenome-assembled contigs were first cut into 10 kbp sections, and then initial raw reads were mapped against these 10 kbp sections before finally binning the sections into MAGs using default settings. Results of CONCOCT binning were visualized by plotting the read-coverage, GC-content, and MAG identity for each contig using ggplot in R v.4.0.3 (Wickham 2009; R Development Core Team 2019).

The CONCOCT results and BlobTools identifications for each metagenome were overlapped to detect which MAGs had been identified as *Ascomycota* by BlobTools. In most cases, the mycobiont genome formed a linear cloud of contigs with 30–50% GC-content at a consistent coverage, a pattern also observed in other lichen metagenome studies (McKenzie et al. 2020; Tagirdzhanova et al. 2021). CONCOCT tended to split the linear cloud into one large metagenome bin at ∼50% GC-content and a series of smaller bins along the “tail” of the linear cloud. These “tail” bins were therefore merged with the large bin to form a single mycobiont MAG, following the example of Tagirdzhanova et al. (2021). In order to remove contaminants, the putative mycobiont MAG was then subjected to a second round of the BlobTools workflow, where any contigs that did not have a top blast hit of either *Ascomycota* or a “no hit” at all were removed. This was done using the BlobTools blobDB.bestsum.table.txt file as input for the SeqKit's grep function (Shen et al. 2016). Supplementary fig. S1, Supplementary Material online, gives a visual representation of the mycobiont filtering pipeline. With contaminants removed, reads mapping to the remaining mycobiont contigs were extracted using the “outm = filtered_R#.fq.gz” function of BBTools “bbmap” (Bushnell 2014). QUAST and BUSCO were used to assess the quality of the final MAGs as described above.

Finally, redundans v0.14a (Pryszcz and Gabaldón 2016) was used to remove redundant contigs, scaffold the remaining contigs and close gaps. This produced our final *Lecanoromycetes* mycobiont MAGs, henceforth referred to as mycobiont genomes. These newly produced mycobiont genomes were then combined with 21 already published Lecanoromycetes mycobiont genomes downloaded from NCBI and Mycocosm (see supplementary table S2, Supplementary Material online, for details). All subsequent analyses were performed on the 45-genome combined dataset of our 24 newly sequenced genomes and the 21 downloaded genomes.

### Mycobiont Gene Prediction and Orthologue Identification

Repeat content libraries were generated de novo for each genome using RepeatModeler v2.0.1, implementing default settings with LTR discovery (Flynn et al. 2020) and then used as a custom library for softmasking with RepeatMasker v4.1.0 (Smit et al. 2015). Gene prediction was performed on the masked assemblies using the funannotate v1.7.0 pipeline (Palmer and Stajich 2019). Funannotate performs gene prediction using Augustus (Stanke et al. 2006), GlimmerHMM (Majoros et al. 2004), and SNAP (Korf 2004) and consolidates them into a single set using EvidenceModeler (Haas et al. 2008). Expressed sequence tags (ESTs) and protein models from the genomes of *Xanthoria parietina*, *Cladonia grayi*, and *Usnea florida* were downloaded from JGI Mycocosm (Grigoriev et al. 2014; Armaleo et al. 2019) (supplementary table S2, Supplementary Material online) and provided to EvidenceModeler to further inform gene prediction. Repeat masking and gene prediction were not performed on the Mycocosm data as this information was already available. Orthologous genes (referred to here as orthogroups) were identified from amino acid sequences of the total 45 genomes using Orthofinder v2.4.0 with default settings (Emms and Kelly 2019). Single-copy orthogroups present in at least 75% of the taxa were used for subsequent tree reconstruction producing a final dataset of 2,214 orthogroups.

### Lecanoromycetes Phylogeny

The amino acid sequences for each of the 2,214 orthogroups were aligned using MAFFT v7.271 with the “—auto” option (Katoh et al. 2009). Ambiguously aligned regions were trimmed using TrimAl v1.4.rev12 with the “—automated1” option (Capella-Gutiérrez et al. 2009). Concatenation and coalescent-based approaches were compared to produce the *Lecanoromycetes* species tree. IQTree v2.0.6 (Nguyen et al. 2015) was used to produce a concatenated alignment of the trimmed MSAs. The optimal amino acid substitution model for each orthogroup was identified using inbuilt tool ModelFinder (Kalyaanamoorthy et al. 2017) and model parameters were estimated under an edge-linked partition scheme with a free evolutionary rate and each orthogroup treated as a separate partition. A ML tree was then produced in IQTree using the edge-linked partition model and branch support was assessed with 1,000 ultrafast bootstrap replicates (UFBoot) (Nguyen et al. 2015; Hoang et al. 2018). IQTree was also used to infer the 2,214 individual ML gene trees, again using ModelFinder. A coalescent-based species tree was reconstructed with the individual orthogroup trees using ASTRAL-III v5.7.1 (Zhang et al. 2018). Bipartition support was assessed via local posterior probabilities (LPP, “-t 3” option in ASTRAL; Sayyari and Mirarab 2016). Genealogical concordance was assessed using gene concordance factors (gCF) and site concordance factors (sCF) calculated in IQTree (Minh et al. 2020).

### Secondary Metabolite Gene Cluster Analysis

Secondary metabolite BGCs were predicted from the mycobiont proteomes using the command line implementation of antiSMASH v5.1.2 (Blin et al. 2019). Homology between gene clusters was inferred by clustering antiSMASH BGCs into BGCFs using BiG-SCAPE (Navarro-Muñoz et al. 2020). BiG-SCAPE calculates pairwise distances between BGCs using a combination of protein domain content, sequence identity, domain order and copy number information. It then links all pairs of BGCs with a distance score below a set cutoff into a sequence similarity network and then performs affinity clustering on each group of connected nodes to assign BGCs to families. We used a range of 0.3–1.0 BiG-SCAPE cutoff values (default-maximum) in our comparisons. BGCs from the MIBiG database v1.4 (Medema et al. 2015) were included in the BiG-SCAPE analysis to link our mycobiont BGCs to published BGCs with known metabolite products. Following Theobald et al. (2018), we chose the minimum cutoff that ensured the MIBiG clusters for the highly similar compounds TAN-1612 and asperthecin were grouped into the same family. This led to a final cutoff of 0.46. BiG-SCAPE was run with the “—hybrids-off” option to ensure that each BGC only appeared in one family. Ribosomally synthesized and post-translationally modified peptide (RiPPs) and saccharide clusters were excluded to reduce computational time.

We estimated turnover in BGC content between the genomes using beta diversity (Baselga 2010), which in this context represents the extent to which dissimilarity in BGC content is due to each genome containing unique sets of BGCs rather than the less diverse genomes simply being subsets of the more diverse ones.

We observed whether BGCF presence–absence patterns reflected phylogenetic relatedness between taxa using PCoA and then tested this using a Mantel test and permutational analysis of variance (PERMANOVA). Firstly, we converted the ML tree into a phylogenetic distance matrix using the cophenetic.phylo function of the R package ape v5.6–1 (Paradis and Schliep 2019). Subsequently, the BiG-SCAPE BGCF presence–absence matrix (excluding nonlichen MIBiG clusters) was transformed into a dissimilarity matrix by calculating Jaccard dissimilarity indices using the “vegdist” function of the R package vegan v.2.5-7. The BGCF dissimilarity matrix was used as input for the PCoA using vegan's “wcmdscale” function. Correlation between the phylogenetic and BGCF distance matrices was tested using a Mantel test implemented with vegan. For PERMANOVA we followed the approach of Mesny and Vannier (2020); to summarize, a principal component analysis (PCA) of phylogenetic distances was performed using the function PCA from the Python package scikit-learn v0.21.3 (Python v3.7.4). The first two principal components (PC1 and 2) were sufficient to cover over 80% of the variance due to phylogeny and were used alongside the Jaccard BGCF distance matrix for PERMANOVA analysis using the adonis2 function of vegan with the model BGCF_DistanceMatrix ∼ phylogeneticPC1 + phylogeneticPC2 (Mesny and Vannier 2020).

Putative anthraquinone gene clusters were identified by searching for families that contained experimentally identified anthraquinone clusters from MIBiG. For families containing these MIBiG clusters, we further investigated PKS evolution by extracting the core PKS enzyme gene from each BGC. We identified which orthogroups these proteins belonged to and pulled all the amino acid sequences belonging to that orthogroup. Amino acid sequences were aligned using MAFFT and poorly aligned sequences were detected using GUIDANCE2 (Sela et al. 2015). These sequences were removed from the original multifasta file and amino acids were aligned again. The multiple sequence alignment was converted into nucleotides using PAL2NAL (Suyama et al. 2006) and a ML gene tree was then reconstructed using RAxML-ng with a GTR + G substitution model (Kozlov et al. 2019). Branch support was assessed with Felsenstein's bootstraps and the Transfer Bootstrap Expectation metric (Lemoine et al. 2018). Bootstrapping convergence was tested using the autoMRE criterion within RAxML-ng.

We then performed functional annotation on the amino acids of the orthogroup. We classified proteins using Pfam domains in the InterPro database (Blum et al. 2021), via InterProscan (https://github.com/ebi-pf-team/interproscan). InterProscan also includes PANTHER analyses that suggest protein function. In addition to the core PKS enzyme genes, we searched for other genes that were potentially shared among all our putative anthraquinone clusters but absent from nonanthraquinone BGCs. These additional genes were functionally annotated in the same way.

Finally, we analyzed the PT domains of the core PKSs to provide further evidence as to whether our putative anthraquinone clusters could synthesize anthraquinones or not. The PT domain is essential for cyclization of the final polyketide and is a key determiner of the final metabolite structure (Crawford et al. 2009). We extracted the PT domain from our PKSs with BLASTp (Camacho et al. 2009), using the asperthecin AptA PT domain from *Aspergillus nidulans* as a query (Szewczyk et al. 2008). Our PT domains were combined with PT domains from a curated dataset of fungal nonreducing PKSs (NR-PKSs) with confirmed metabolite products from (Liu et al. 2015), which were downloaded from NCBI. The PT domains were then aligned to the Pfam seed alignment for the polyketide PT domain (*PS-DH* PF14765: https://www.ebi.ac.uk/interpro/entry/pfam/PF14765/) using a HMM alignment via the following perl script: https://github.com/reubwn/scripts/blob/master/hmmsearch-easy.pl. Pfam seed alignments reflect the diversity of a particular protein domain across the tree of life and thus help to place our potential anthraquinone genes within a wider evolutionary context. We then reconstructed a ML tree from the resulting alignment using IQTree with 1,000 ultrafast bootstraps and ModelFinder to determine the optimal substitution model. All bioinformatics scripts for our pipeline can be found here: https://github.com/theo-llewellyn/TeloschistalesMetagenomics.

## Supplementary Material

evad002_Supplementary_Data

## Data Availability

The genomic data underlying this article are available in the GenBank Nucleotide Database under the BioProject accession PRJNA783777. Individual genome accession numbers are shown in table 1. Genomes downloaded from the public domain are shown in supplementary table S2, Supplementary Material online, along with their relevant databases and accession numbers. Additional data files for anthraquinone PKS amino acid and nucleotide alignments, single-copy orthogroup alignments, raw phylogenetic gene and species trees and HMM seed alignment and tree are deposited in Zenodo doi:10.5281/zenodo.7015195. All bioinformatic scripts used to generate results can be found here: https://github.com/theo-llewellyn/TeloschistalesMetagenomics.
