# Supplementary material for: Metagenomics Shines Light on the Evolution of “Sunscreen” Pigment Metabolism in the Teloschistales (Lichen-Forming Ascomycota)
Source: Genome Biol Evol. 2023 Jan 12;15(2):evad002. doi: 10.1093/gbe/evad002 (PMC9907504; doi:10.1093/gbe/evad002)
Supplement: evad002_Supplementary_Data [file evad002_supplementary_data.pdf]

Supplementary materials

Supplementary Table S1: Collection information for newly sequenced lichens in this study

Taxon	Locality	Legit	Collection date
Diploschistes diacapsis	Catalunya, Spain	XL, AA	04.09.2018
Umbilicaria vellea	Catalunya, Spain	XL, AA	22.09.2018
Letrouitia transgressa	Sri Lanka	AA	31.05.2017
Letrouitia transgressa (2)	Sri Lanka	AA	26.04.2017
Teloschistes flavicans	Devon, UK	TBL	16.10.2019
T. chrysophthalmus	Catalunya, Spain	XL, AA	02.04.2019
Teloschistes peruensis	Santiago, Peru	OW-1716	20.01.2015
Xanthomendoza fulva	Catalunya, Spain	XL, AA	20.09.2018
Flavoplaca oasis	Negev Desert, Israel	MT	12.11.2018
Xanthoria sp.	Telve, Italy	TBL	08.09.2018
Xanthoria sp. 2	Belluno, Italy	TBL	01.09.2019
Xanthoria aureola	Catalunya, Spain	XL, AA	No date
Xanthoria steineri	Negev Desert, Israel	MT	04.12.2018
Xanthoria mediterranea	Negev Desert, Israel	MT	05.12.2018
Gyalolechia ehrenbergii	Negev Desert, Israel	MT	12.11.2018
Letrouitia leprolyta	Udawalawa, Sri Lanka	AA	No date
Usnochroma carphinea	Catalunya, Spain	XL, AA	14.04.2019
Caloplaca ligustica	Catalunya, Spain	XL	16.04.2019
Caloplaca aetnensis	Catalunya, Spain	XL, AA	16.04.2019
Caloplaca aegea	Catalunya, Spain	XL, AA	29.10.2018
Variospora aurantia	Negev Desert, Israel	MT	04.12.2018
Seirophora villosa	Negev Desert, Israel	MT	04.12.2018
Seirophora lacunosa	Negev Desert, Israel	MT	12.11.2018
Seirophora lacunosa (2)	Catalunya, Spain	AA, XL	20.05.2019

Supplementary Table S2: Lichen genome assemblies in the custom *Lecanoromycetes* database used in BLAST and phylogenomics and comparative genomics analysis. N50 = contig N50. * = assembly from metagenomic data. Numbers in parentheses after species names indicate multiple assemblies of same taxon. † = *Teloschistaceae*

Species	Genome size (Mbp)	N50 (kb)	Database	Accession number
*Alectoria sarmentosa**	39.96	93	GenBank	GCA_009733775.1
Cladonia macilenta	37.12	1469	GenBank	GCA_000444155.1
Cladonia metacorallifera	36.68	1591	GenBank	GCA_000482085.2
Cladonia rangiferina	35.67	273	GenBank	GCA_006146055.1
Cladonia uncialis	32.85	35	GenBank	GCA_002927785.1
Cladonia grayii	34.62	243	JGI Mycocosm	Cgr/DA2myc/ss v1.0
Cyanodermella asteris	28.67	1790	GenBank	GCA_900618795.1
Evernia prunsatri	40.35	264	GenBank	GCA_003184365.1
Gyalolechia flavorubscens[†]	34.47	1693	GenBank	GCA_000442125.1
Lasallia hispanica	41.21	145	GenBank	GCA_003254425.1
Lasallia pustulata	32.91	1808	GenBank	GCA_008636195.1
Lasallia pustulata (2)	39.23	21	GenBank	GCA_900169345.1
Lasallia pustulata (3)	39.23	21	GenBank	GCA_000938525.1
*Letharia columbiana**	52.27	667	GenBank	GCA_014066305.1
*Letharia lupina**	49.20	2098	GenBank	GCA_014066315.1
Pseudevernia furfuracea	37.80	1178	GenBank	GCA_003184345.1
Ramalina intermedia	26.25	273	GenBank	GCA_003073195.1
Ramalina peruensis	26.99	40	GenBank	GCA_001956345.1
Rusavskia elegans[†]	44.32	386	GenBank	GCA_011316305.1
Usnea florida	44.32	502	JGI Mycocosm	ATCC18376 v1.0
Usnea hakonensis	41.20	166	GenBank	GCA_013423325.1
Umbilicaria muehlenbergii	34.81	7009	GenBank	GCA_000611775.1
Xanthoria parietina[†]	31.90	1731	JGI Mycocosm	46-1-SA22 v1.1

3

Supplementary Figure S1: An example of mycobiont reads filtering combining BlobTools and CONCOCT methods for the lichen metagenome data of *Xanthoria mediterranea* (*Teloschistaceae*, Ascomycota). (*a*) Blobplot pre-mycobiont filtering showing the GC proportion against coverage for each contig in the metagenome assembly. Histograms for both axes are shown. Circle colour represents taxonomic identification via BLAST/DIAMOND. Circle diameter represents contig length. Legend shows contig count, total span (Megabases) and N50 for each taxon. Light grey circles are contigs that could not be taxonomically identified. (*b*) Plot showing GC proportion against coverage for the same metagenome as in (*a*). Contigs (points) are coloured by the CONCOCT metagenome bin to which they were assigned and labelled accordingly with a unique bin number. (*c*) Facet plot showing GC proportion against coverage for each CONCOCT bin individually. Bin numbers are shown above each facet and contigs belonging to that bin are highlighted (coloured points) against the full metagenome assembly (light grey points). Points are coloured by summarised BlobTools taxonomic ID: orange = *Ascomycota*, green = Bacteria, purple = Chlorophyta, blue = no hit, dark grey = other. (*d*) Blobplot after merging CONCOCT bins 14, 50, 65, 78, and 80 and removing remaining non-mycobiont reads. Colour coding for each subplot is unique and not comparable between subplots.

Supplementary Figure S2: Violin plots comparing post-assembly summary statistics of published lichenised fungal genomes from pure mycobiont culture (orange) with lichenised fungal genomes from metagenomic data (yellow). Top row from left to right: Percentage complete BUSCO genes, N50 score in 1000s of bases (K), Number of assembly scaffolds. Bottom row: Assembly size in megabase pairs (Mbp), Number of predicted genes.

Supplementary Figure S3: Scatterplots showing the relationship between number of detected BGCs per genome and assembly fragmentation (N50) for 50 lichen mycobiont genome assemblies. Dataset includes the 45 assemblies shown in Supplementary Tables 1 and 2 plus five additional long-read genomes from the following publications (Allen et al. 2021; Wilken et al. 2020; Singh et al. 2021; Gerasimova et al. 2022). (*a*) Number of BGCs shows no significant relationship with N50, partial F-test at a $p < 0.05$ significance level ($F_{48} = 2.9819$, $p = 0.09063$). (*b*) Same as (a) except for removal of a potential outlier *Umbilicaria muehlenbergii* which had a significantly higher N50 than other assemblies. Again, number of BGCs shows no significant relationship with N50 ($F_{47} = 0.8684$, $p = 0.3562$). Sequencing technology (long-read or short-read) is highlighted by dot colour. Fitted regression lines and 95% confidence intervals (coloured boxes around regression lines) were predicted using the linear model.

Supplementary Figure S4: Results of Interpro functional analysis performed with PANTHER to annotate the gene sequences on the orthogroup containing our putative anthraquinone PKSs. (*a*) Waffle chart representation of PKS protein PANTHER functional annotations. Each square represents a PKS protein in the orthogroup and is coloured by the assigned PANTHER function from Interpro analysis. (*b*) Orthofinder gene tree for the orthogroup (shown in Figure 3) with PANTHER functional annotation plotted as coloured tip dots. Colours follow the key in *(a*). Our putative anthraquinone PKSs clade is highlighted in the grey block.

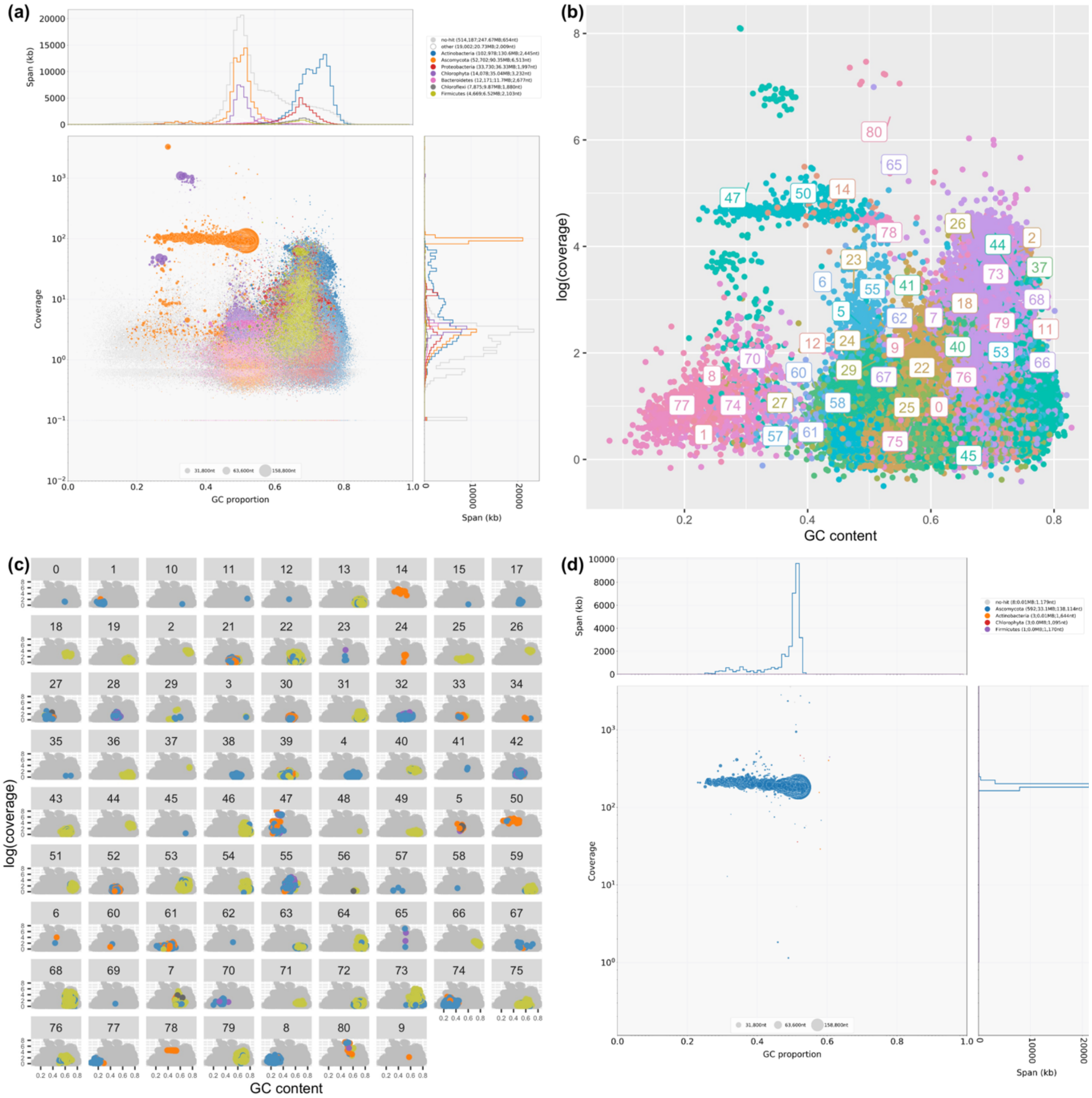
(a)
Span (kb)
Coverage
GC proportion
Span (kb)
(b)
log(coverage)
GC content
(c)
log(coverage)
GC content
(d)
Span (kb)
Coverage
GC proportion
Span (kb)

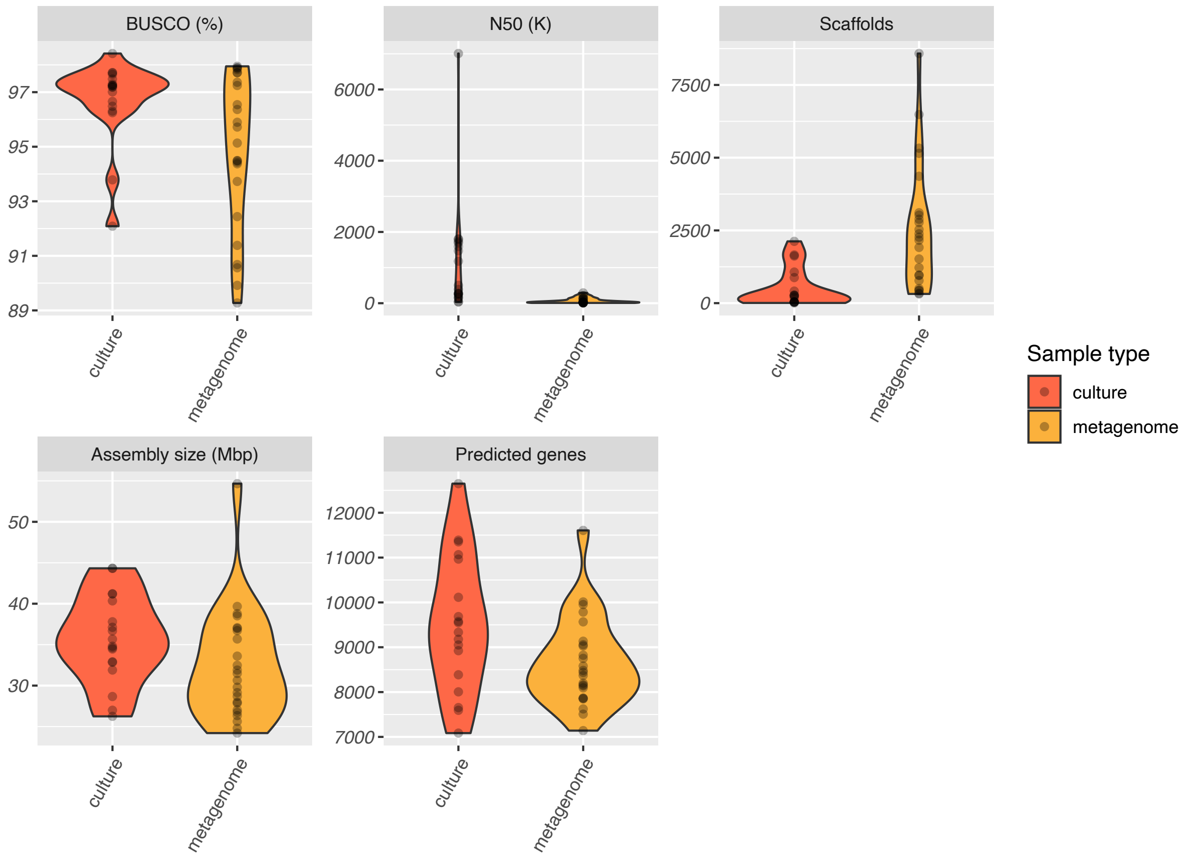

BUSCO (%)
97
95
93
91
89
culture
metagenome
N50 (K)
6000
4000
2000
0
culture
metagenome
Scaffolds
7500
5000
2500
0
culture
metagenome
Sample type
culture
metagenome
Assembly size (Mbp)
50
40
30
culture
metagenome
Predicted genes
12000
11000
10000
9000
8000
7000
culture
metagenome

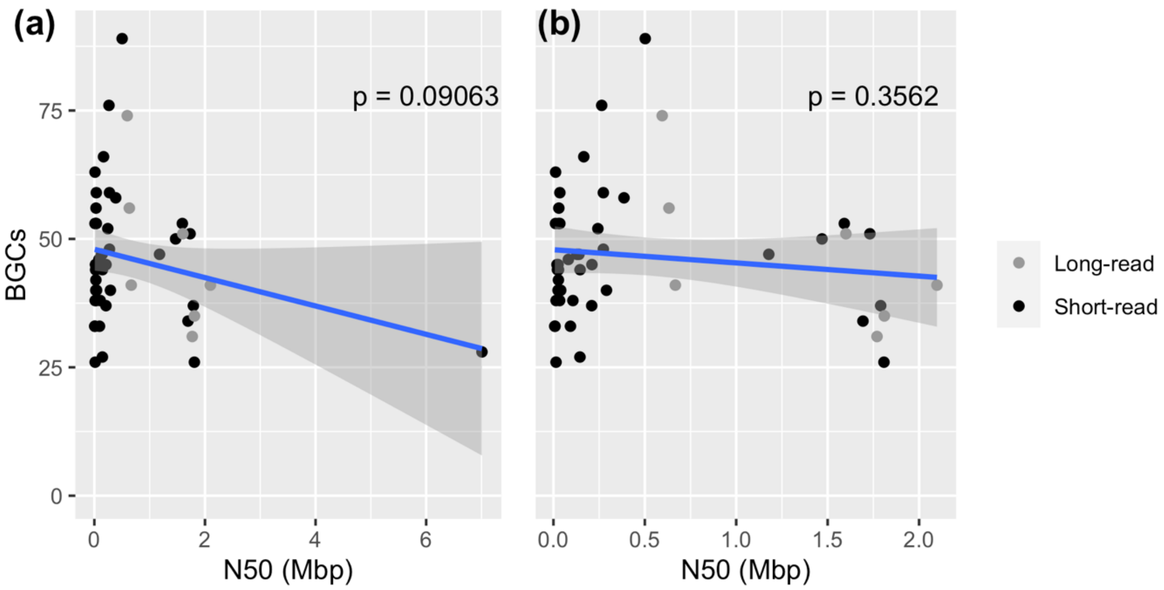
(a)
(b)
p = 0.09063
p = 0.3562
BGCs
N50 (Mbp)
N50 (Mbp)
Long-read
Short-read

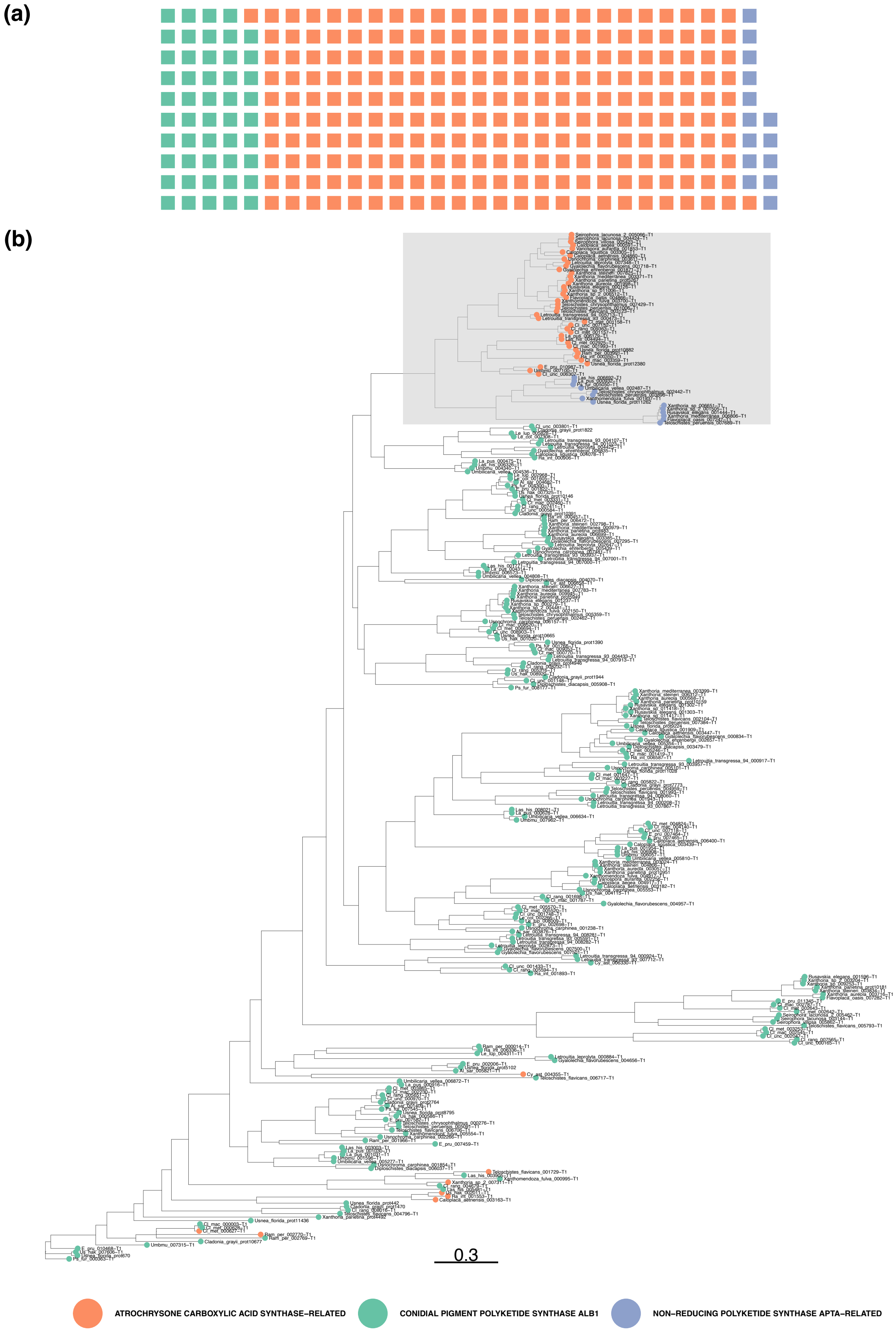
(a)
(b)
0.3
ATROCHRYSONE CARBOXYLIC ACID SYNTHASE–RELATED
CONIDIAL PIGMENT POLYKETIDE SYNTHASE ALB1
NON–REDUCING POLYKETIDE SYNTHASE APTA–RELATED

53
